# Efficiency of a Constrained Linear Genomic Selection Index To Predict the Net Genetic Merit in Plants

**DOI:** 10.1534/g3.119.400677

**Published:** 2019-09-30

**Authors:** J. Jesus Cerón-Rojas, Jose Crossa

**Affiliations:** Biometrics and Statistics Unit, International Maize and Wheat Improvement Center (CIMMYT), Apdo. Postal 6-641, 06600, Mexico City, Mexico

**Keywords:** Genomic estimated breeding value, Selection response, Expected genetic gain per trait, Molecular marker effects, GenPred, Shared Data Resources, Genomic Prediction

## Abstract

The constrained linear genomic selection index (CLGSI) is a linear combination of genomic estimated breeding values useful for predicting the net genetic merit, which in turn is a linear combination of true unobservable breeding values of the traits weighted by their respective economic values. The CLGSI is the most general genomic index and allows imposing constraints on the expected genetic gain per trait to make some traits change their mean values based on a predetermined level, while the rest of them remain without restrictions. In addition, it includes the unconstrained linear genomic index as a particular case. Using two real datasets and simulated data for seven selection cycles, we compared the theoretical results of the CLGSI with the theoretical results of the constrained linear phenotypic selection index (CLPSI). The criteria used to compare CLGSI *vs.* CLPSI efficiency were the estimated expected genetic gain per trait values, the selection response, and the interval between selection cycles. The results indicated that because the interval between selection cycles is shorter for the CLGSI than for the CLPSI, CLGSI is more efficient than CLPSI per unit of time, but its efficiency could be lower per selection cycle. Thus, CLGSI is a good option for performing genomic selection when there are genotyped candidates for selection.

The unconstrained linear genomic selection index (LGSI) is a linear combination of genomic estimated breeding values (GEBVs) and was originally proposed by Togashi *et al.* (2011); however, Ceron-Rojas *et al.* (2015) developed the LGSI theory completely and applied the LGSI theoretical results to real and simulated data. The LGSI is useful for predicting the net genetic merit, which in turn is a linear combination of true unobservable breeding values of the traits weighted by their respective economic values (Hazel 1943). In LGSI, all marker effects of the genotyped individuals in the training population are estimated using marker and phenotypic data; these estimated effects are then used in subsequent selection cycles to obtain GEBVs that are predictors of the individual breeding values in the testing population for which there is only marker information about the candidates for selection. In LGSI, the GEBVs can be obtained by multiplying the genomic best linear unbiased predictor (GBLUP) of the estimated marker effects in the training population (VanRaden 2008) by the coded marker values obtained in the testing population in each selection cycle. Applying LGSI in plant or animal breeding requires genotyping the candidates for selection to obtain marker effects and GEBVs, and then predicting and ranking the net genetic merit of the candidates for selection.

The LGSI was developed in the genomic selection (GS) context in which animals and plants are selected based on the GEBV of the candidates for selection. Meuwissen *et al.* (2001) developed the GS theory and showed that it increases the accuracy of predicting the breeding values of the candidates for selection, and reduces the intervals between selection cycles and the costs of the breeding programs. Meuwissen *et al.* (2001) suggested estimating all marker effects jointly using linear mixed models and Bayesian methods to increase the accuracy of the predicted breeding values because many genes affect the quantitative traits, which are of most concern to plant and animal breeders. Isik *et al.* (2017), however, have indicated that GS has not changed the fundamental basis of breeding methods, since in GS the individuals are ranked based on their estimated breeding values, which has been the selection method used by animal and plant breeders for a long time. Nevertheless, because GS decreases the generation interval, it leads to a much higher genetic gain per year. For example, in the plant breeding context, a four-year breeding cycle, which includes three years of field testing, can be reduced to only four months, *i.e.*, the time required to grow and cross plants (Lorenz *et al.* 2011). Börner and Reinsch (2012) have indicated that GS could replace traditional progeny testing when maximizing the genetic gain per year, as long as the accuracy of GEBV is higher than or equal to 0.45.

One of the main problems associated with the LGSI theory is that the values of its expected genetic gain per trait (or multi-trait selection response) can increase or decrease in a positive or negative direction without control. In the phenotypic selection context, Kempthorne and Nordskog (1959) developed a restricted linear selection index that allows imposing restrictions equal to zero on the expected genetic gain of some traits. Other authors (Mallard 1972; Harville 1975; Tallis 1985) extended the Kempthorne and Nordskog (1959) approach and developed a constrained linear phenotypic selection index (CLPSI) that attempts to make some traits change their expected genetic gain values based on a predetermined level, while the rest of the traits remain without restrictions. Itoh and Yamada (1987) showed that the Mallard (1972), Harville (1975) and Tallis (1985) indices give the same results. The CLPSI is the most general index and includes the unconstrained and restricted phenotypic indices as particular cases.

Céron-Rojas and Crossa (2018, Chapter 3) developed a constrained linear genomic selection index (CLGSI); however, these authors did not evaluate this index completely. For example, they did not give results associated with the CLGSI expected genetic gain per trait, which is the main parameter of this index because the breeder imposes constraints on it to make some traits change their expected genetic gain values based on a predetermined level, while the rest of the traits remain without restrictions.

This study had two main objectives: first, to apply the CLGSI theoretical results to two real datasets and to seven simulated datasets using only GEBVs for selecting non-phenotyped candidates for selection, and second, to compare the relative efficiency of CLGSI and CLPSI using real and simulated datasets in a single-stage context. The criteria we used to compare CLGSI *vs.* CLPSI efficiency were the estimated expected genetic gain per trait values, the selection response, and the interval between selection cycles. One additional criterion was that the CLGSI and CLPSI proportional constant value should be positive (Céron-Rojas and Crossa 2018, Chapters 3 and 6 for details). The results indicated that because the interval between selection cycles was shorter for CLGSI than for CLPSI, CLGSI is more efficient than CLPSI per unit of time, but its efficiency could be lower per selection cycle.

We applied the CLGSI theory assuming that the GEBV values have multivariate normal distribution and that the CLGSI (CLPSI) and the net genetic merit have joint bivariate normal distribution. Under this last assumption, the regression of the net genetic merit on any linear function of the phenotypic or GEBV values is linear (Kempthorne and Nordskog 1959).

## Material and Methods

### Objectives of the constrained linear selection index

Cerón-Rojas *et al.* (2016) and Céron-Rojas and Crossa (2018, Chapter 3) described the CLPSI theory; thus, in this work, we shall describe only the CLGSI theory. Let ${µ}_{i}$ be the population mean of the *i*^th^ trait before selection. One of the main CLGSI objectives is to change ${µ}_{i}$ to ${µ}_{i}+d_{i}$, where $d_{i}$ is the *i*^th^ (*i* = 1, 2, …, *r*; *r* = number of constrained traits) trait constraint or predetermined proportional gain imposed by the breeder on the CLGSI expected genetic gain per trait. Additional CLGSI objectives are to maximize the selection response; to predict the net genetic merit (H=w′g, where $w\prime =\left[\begin{array}{cc} {\text{w}}_{1} & {\text{w}}_{2} \end{array}\;\;\;\begin{array}{cc} \ldots & {\text{w}}_{t} \end{array}\right]$ and $g^{\prime }=\left[\begin{array}{cc} {\text{g}}_{1} & {\text{g}}_{2} \end{array}\;\;\;\begin{array}{cc} \ldots & {\text{g}}_{t} \end{array}\right]$ are 1×t (t=number of traits) vectors of economic weights and true unobservable breeding values, respectively); to select individuals with the highest H values as parents of the next generation; and to provide the breeder with an objective rule for evaluating and selecting several traits simultaneously.

### The constrained linear genomic selection index

Let $z^{\prime }=[\begin{array}{cccc} z_{1} & z_{2} & \cdots & z_{t} \end{array}]$ be a vector of genomic breeding values for t traits (**Appendix 1**, Equations A1 to A4 for details). The CLGSI used to predict the individual net genetic merit of a candidate for selection is

$$I=\beta_{1}z_{1}+\beta_{2}z_{2}+\ldots +\beta_{t}z_{t}=\beta^{'}z,$$ (1)

where $\beta \prime =\left[\begin{array}{cc} \beta_{1} & \beta_{2} \end{array}\;\;\;\begin{array}{cc} \ldots & \beta_{t} \end{array}\right]$ is the CLGSI vector of coefficients and $z_{i}$ (*i*= 1, 2, …, t) is the genomic value associated with trait *i*^th^. Ceron-Rojas *et al.* (2015) described Equation (1) in the unconstrained LGSI.

### The CLGSI selection response

The CLGSI selection response (R) can be written as

$$R=\frac{1}{L}k\sigma_{H}\rho_{HI},$$ (2)

where k is the selection intensity, *L* denotes the interval between selection cycles, $\sigma_{H}=\sqrt{w\prime Cw}$ is the standard deviation of H=w′g, Var(g)=C is the covariance matrix of **g**, and $\rho_{{}_{HI}}=\frac{w^{\prime }\Gamma \beta }{\sqrt{w^{\prime }Cw}\sqrt{\beta^{\prime }\Gamma \beta }}$ is the correlation between $H=w^{\prime }g$ and $I=\beta^{'}z$, where $w^{\prime }\Gamma \beta =\sigma_{HI}$ is the covariance between $H=w^{\prime }g$ and $I=\beta^{'}z$, **Γ=Var(z)** is the covariance matrix of genomic breeding values (**Appendix 1**, Equation A2 for details), and $\sigma_{I}=\sqrt{\beta^{\prime }\Gamma \beta }$ is the standard deviation of $I=\beta^{'}z$. Equation (1) indicates that the genetic gain that can be achieved in R by selecting for several traits simultaneously within a population of plants, is the product of k, $\sigma_{H}$ and $\rho_{HI}$ (Kempthorne and Nordskog 1959). Thus, to increase selection progress, $\rho_{HI}$ should be as large as possible. Céron-Rojas and Crossa (2018, Chapter 3) described the selection response of the CLPSI, which is very similar to Equation (2).

### The CLGSI expected genetic gain per trait

The CLGSI expected genetic gain per trait (E, or multi-trait selection response) is the covariance between the genomic breeding value vector $z^{\prime }$ and the index, $I=\beta^{'}z$ (Equation 1), weighted by the standard deviation of I, $\sigma_{I}=\sqrt{\beta^{\prime }\Gamma \beta }$, multiplied by the selection intensity (k) and divided by the interval between selection cycles (*L*), *i.e.*,

$$E=\frac{k}{L}\frac{\Gamma \beta }{\sigma_{I}}.$$ (3)

Equation (3) is a t×1 vector, *i.e.*, $E^{\prime }=[\begin{array}{cccc} E_{1} & E_{2} & ... & E_{t} \end{array}]$, where $E_{i}=\frac{k}{L\sigma_{I}}Cov(I,z_{i})=\frac{k}{L\sigma_{I}}[\beta_{1}\sigma_{1i}+\beta_{2}\sigma_{2i}+\cdots +\beta_{j}\sigma_{ji}^{}+\cdots +\beta_{t}\sigma_{ti}]$ (i= 1, 2, …,t) is the expected genetic gain of trait *i*^th^, $z_{i}$ is the *i*^th^ genomic breeding value, $I=\sum_{j=1}^{t}\beta_{j}z_{j}$ is the index value (Equation 1) for one individual, $Cov(I,z_{i})$ is the covariance between I and $z_{i}$, and $\sigma_{ji}$ is the covariance between $z_{i}$ and the j^th^ (j= 1, 2, …,t) index genomic breeding value. We defined all the other terms of Equation (3) in Equation (2).

### The CLGSI vector of coefficients

Maximizing $\rho_{HI}$ (Equation 2) is equivalent to minimizing the mean squared difference between the net genetic merit H=w′g and the index $I=\beta^{'}z$, *i.e.*, $E[{\left(H-I\right)}^{2}]$, with respect to the vector of coefficients β under the restriction that Equation (3) values are equal to the $d_{i}$ values (*i* = 1, 2, …, *r*) imposed by the breeder. Details of the process used to obtain the CLGSI vector of coefficients (β) are given in **Appendix 2** (Equations A6 and A7). Here we present only the main result. The CLGSI vector of coefficients is

β=Kw, (4a)

where $w\prime =\left[\begin{array}{cc} {\text{w}}_{1} & {\text{w}}_{2} \end{array}\;\;\;\begin{array}{cc} \ldots & {\text{w}}_{t} \end{array}\right]$ is a vector of economic weights, $K=[I_{t}-Q]$, $Q=UD{\left(D^{\prime }U^{\prime }\Gamma UD\right)}^{-1}D^{\prime }U^{\prime }\Gamma$, $I_{t}$ is an identity matrix of size t×t, D is a Mallard (1972) matrix described in **Appendix 2**, and U is the Kempthorne and Nordskog (1959) matrix described in **Appendix 3**. When D=U ($d_{i}=0$ for all traits), matrix K can be written as $K_{G}=[I_{t}-Q_{G}]$, where $Q_{G}=U{\left(U^{\prime }\Gamma U\right)}^{-1}U^{\prime }\Gamma$. In this last case, the CLGSI is a null restricted LGSI similar to the Kempthorne and Nordskog (1959) restricted index. When D=U and $U^{\prime }$ is a null matrix, β=w, the vector of economic weights or the unconstrained LGSI vector of coefficients (Ceron-Rojas *et al.* 2015). Thus, the CLGSI is more general than the LGSI and includes the null restricted and the unrestricted LGSI as particular cases. The vector of coefficients β should maximize the selection response (R, Equation 2) and make Equation (3) values similar to $d_{i}$ values.

It is possible to show (Itoh and Yamada 1987; Céron-Rojas and Crossa 2018, Chapter 3) that another way of writing Equation (4a) is

$$\beta =K_{G}w+\theta U{\left(U^{\prime }\Gamma U\right)}^{-1}d,$$ (4b)

where $\theta =\frac{d^{\prime }{\left(U^{\prime }\Gamma U\right)}^{-1}U^{\prime }\Gamma w}{d^{\prime }{\left(U^{\prime }\Gamma U\right)}^{-1}d}$ is the proportionality constant and $d^{\prime }=[\begin{array}{cccc} d_{1} & d_{2} & ... & d_{r} \end{array}]$ is the vector of the constraints imposed by the breeder. According to Itoh and Yamada (1987), θ should be higher than zero (θ>0) because when θ>0, the CLGSI moves the population means in the opposite direction to the predetermined desired direction. In addition, when θ=0, we would have the CLGSI with null constraints similar to the Kempthorne and Nordskog (1959) restricted index. Thus, θ is a good criterion for determining when the CLGSI moves the population means in the desired direction, which will occur when θ>0. While Equation (4a) is associated with the Mallard (1972) constrained index, Equation (4b) is associated with the Tallis (1985) constrained index; however, Equations (4a) and (4b) express the same result in a different mathematical way.

In **Appendix 1** (Equation A5), we give a method for estimating matrix Γ, and in **Appendix 2** (Equation A8), we explain how to estimate β.

### Maximized CLGSI selection response and optimized expected genetic gain per trait

The maximized CLGSI selection response is

$$R=k\sqrt{\beta^{\prime }\Gamma \beta },$$ (5)

while the optimized CLGSI expected genetic gain per trait is the same as Equation (3). In Equation (5) we omitted *L* to simplify notation. Whereas in Equation (2) the selection response can take any value, in Equation (5), R gives the maximum value of Equation (2). In **Appendix 2** (Equations A9 and A10), we indicate how to estimate R and E.

### Obtaining the genomic estimated breeding values (GEBV)

Several authors (VanRaden 2008; Ceron-Rojas *et al.* 2015; Isik *et al.* 2017, Chapter 11; Céron-Rojas and Crossa 2018, Chapter 5) have given detailed descriptions of how to obtain trait genomic breeding values (GEBV) which are predictors of trait unobservable breeding values. In CLGSI, we fitted phenotypic and marker data from the training population in a statistical model to estimate all available marker effects; these estimates were then used to obtain GEBV that are predictors of the individual traits’ true genomic breeding values in a testing population for which there is only marker information. We obtained the GEBV in the non-phenotyped testing population by multiplying the estimated marker effects obtained in the training population by the coded marker values obtained in the testing population at each selection cycle.

### Criteria for comparing CLGSI efficiency *vs.* CLPSI efficiency

The criteria used to compare CLGSI *vs.* CLPSI efficiency when making genomic and phenotypic selection were as follows. First, the estimated theta value (θ) should be positive (θ>0). Second, the estimated expected genetic gain values should be close to the constraints ($d_{i}$) imposed by the breeder. Third, the estimated selection response value should be equivalent to the true selection response value. Fourth, the size of the interval between selection cycles (*L*) should be short to increase the selection gain per year.

In this work, we do not consider the problem associated with the cost of obtaining measures of each phenotypic trait and the process of genotyping individual candidates for selection. However, some authors (Schaeffer 2006; Börner and Reinsch 2012) have considered this problem in the animal breeding context.

## Materials

### Real data

We used two real maize (*Zea mays* L.) F2 populations: “JMpop1 DTMA Mexico optimum environment” and “6x1020 WEMA Africa optimum environment” (hereafter we shall refer to the first and second maize F2 populations as dataset 1 and dataset 2, respectively). The training population (C0) of each dataset contains genotypic data and four phenotypic traits: grain yield (GY, t/ha), plant height (PHT, cm), ear height (EHT, cm), and anthesis days (AD, d). In addition, each dataset has three sets of individuals from the training population (C0) and two testing populations (C1 and C2). We present the number of individuals and molecular markers in each population in Table 1. Assuming that the breeding objective was to increase GY while decreasing PHT, EHT, and AD, the vector of economic weights in C0, C1, and C2 for GY, PHT, EHT, and AD was $w^{\prime }=[\begin{array}{ccc} 5 & -0.3 & \begin{array}{cc} -0.3 & -1 \end{array} \end{array}]$ for both indices and the two datasets. For illustration purposes only, in this work we used three selection cycles (C0, C1 and C2) for both datasets to illustrate the theoretical results and the efficiency of CLGSI and CLPSI.

**Table 1:** Two real maize (*Zea mays* L.) ${\text{F}}_{2}$ populations and the number of genotypes (*g*) and molecular markers (*m*) used in three selection cycles (cycles 0, 1 and 2)

	Real datasets			
	Dataset 1		Dataset 2	
Cycle	*g*	*m*	*g*	*m*
0	247	195	181	205
1	320	195	274	205
2	303	195	274	205

For illustration purposes only, to select traits GY, PHT, EHT and AD, we imposed two sets of restrictions. First we restricted traits GY and PHT with vector $d^{\prime }=[0.5\;\;-1.0]$ and matrices $D^{\prime }=[\begin{array}{cc} -1.0 & -0.5 \end{array}]$ and $U^{\prime }=\left[\begin{array}{cccc} 1 & 0 & 0 & 0\\ 0 & 1 & 0 & 0 \end{array}\right]$ (**Appendices 2 and 3** for details), and later, we restricted traits GY, PHT and EHT with vector $d^{\prime }=[\begin{array}{ccc} 0.5 & -1.0 & -0.5 \end{array}]$ and matrices $U^{\prime }=\left[\begin{array}{cccc} 1 & 0 & 0 & 0\\ 0 & 1 & 0 & 0\\ 0 & 0 & 1 & 0 \end{array}\right]$ and $D^{\prime }=\left[\begin{array}{ccc} -0.5 & 0 & -0.5\\ 0 & -0.5 & 1.0 \end{array}\right]$for both datasets and for the two indices (CLGSI and CLPSI). The total proportion (p) of retained value for these datasets was p= 0.10 (k=1.755) for both indices and the two datasets with the two sets of constraints.

### Simulated datasets

The datasets were simulated by Ceron-Rojas *et al.* (2015) with QU-GENE software (Podlich and Cooper 1998) using 2500 molecular markers and 315 quantitative trait loci (QTL) for eight phenotypic selection cycles (C0 to C7), each with four traits ($T_{1}$, $T_{2}$, $T_{3}$ and $T_{4}$), 500 genotypes and 4 replicates for each genotype. The authors distributed the markers uniformly across 10 chromosomes and the QTL randomly across the 10 chromosomes to simulate maize (*Zea mays* L.) populations. A different number of QTL affected each of the four traits: 300, 100, 60, and 40, respectively. The common QTL affecting the traits generated genotypic correlations of -0.5, 0.4, 0.3, -0.3, -0.2, and 0.1 between $T_{1}$ and $T_{2}$, $T_{1}$ and $T_{3}$, $T_{1}$ and $T_{4}$, $T_{2}$ and $T_{3}$, $T_{2}$ and $T_{4}$, $T_{3}$ and $T_{4}$, respectively. The economic weights for $T_{1}$, $T_{2}$, $T_{3}$ and $T_{4}$ were 1, -1, 1 and 1, respectively. Additional details of the simulated data can be seen in Ceron-Rojas *et al.* (2015).

We used seven phenotypic and genomic selection cycles (C1 to C7) with p= 0.10 (k=1.755) in each cycle. We selected all four traits in each selection cycle. For illustration purposes only, to select traits $T_{1}$, $T_{2}$, $T_{3}$ and $T_{4}$, we imposed two sets of restrictions. First we restricted traits $T_{1}$ and $T_{2}$ with vector $d^{\prime }=[5\;\;-2]$ and matrices $U^{\prime }=\left[\begin{array}{cccc} 1 & 0 & 0 & 0\\ 0 & 1 & 0 & 0 \end{array}\right]$ and $D^{\prime }=[\begin{array}{cc} -2 & -5 \end{array}]$, and later, we restricted traits $T_{1}$, $T_{2}$ and $T_{3}$ with vector $d^{\prime }=[\begin{array}{ccc} 5 & -2 & 3 \end{array}]$ and matrices $U^{\prime }=\left[\begin{array}{cccc} 1 & 0 & 0 & 0\\ 0 & 1 & 0 & 0\\ 0 & 0 & 1 & 0 \end{array}\right]$ and $D^{\prime }=\left[\begin{array}{ccc} 3 & 0 & -5\\ 0 & 3 & 2 \end{array}\right]$for both datasets and both indices.

### Real and simulated data availability

The real and simulated datasets are available in the *Application of a Genomics Selection Index to Real and Simulated Data* repository, at http://hdl.handle.net/11529/10199. The two real datasets used in this work are the folder named “File Real_Data_Sets_GSI” that contains four folders called “DATA_SET-3, 4, 5 and 6”. Each of the four folders in turn contains four Excel data files. The four Excel data files within the folder DATA_SET-3 are as follows: DATA_SET-3_Markers_Cycle-0, 1, 2, and DATA_SET-3_Phenotypic_Cycle-0. The first three Excel files contain the marker coded values for cycles 0, 1 and 2, while the Excel file DATA_SET-3_Phenotypic_Cycle-0 contains the phenotypic information of cycle 0 (training population). The Excel data files of the other folders were described in a similar manner as for folder 3. In this work, we used datasets 3 and 6 to make selections and to estimate the theta values, the selection response and the genetic expected gains. The results are presented in Tables 2 and 3.

**Table 2:** Two real datasets for estimated CLGSI*^a^* parameters constructed with four traits (GY, PHT, EHT and AD) for two and three constraints in three selection cycles

	Dataset 1 with two constraints					
		Estimated expected genetic gain per trait				Estimated maximum
Cycle	Theta	GY	PHT	EHT	AD	Response
0	2.32	0.76	−1.51	−4.05	0.71	4.73
1	1.70	0.65	−1.30	−3.47	0.67	4.02
2	1.57	0.61	−1.22	−3.63	0.56	3.96
Mean1*^b^*	1.86	0.67	−1.35	−3.72	0.65	4.24
Mean2*^c^*	—	0.45	−0.90	−2.48	0.43	2.82
	Dataset 1 with three constraints					
		Estimated expected genetic gain per trait				Estimated maximum
Cycle	Theta	GY	PHT	EHT	AD	Response
0	2.74	0.84	−1.68	−0.84	0.43	5.02
1	1.99	0.72	−1.44	−0.72	0.41	4.27
2	1.89	0.70	−1.39	−0.70	0.36	4.18
Mean1*^b^*	2.21	0.75	−1.50	−0.75	0.40	4.49
Mean2*^c^*	—	0.50	−1.00	−0.50	0.27	2.99
	Dataset 2 with two constraints					
		Estimated expected genetic gain per trait				Estimated maximum
Cycle	Theta	GY	PHT	EHT	AD	Response
0	0.84	0.42	−0.83	−1.30	−0.36	3.08
1	0.76	0.43	−0.86	−0.61	−0.14	2.73
2	0.68	0.41	−0.82	−0.43	−0.11	2.54
Mean1*^b^*	0.76	0.42	−0.84	−0.78	−0.20	2.79
Mean2*^c^*	—	0.28	−0.56	−0.52	−0.13	1.86
	Dataset 2 with three constraints					
		Estimated expected genetic gain per trait				Estimated maximum
Cycle	Theta	GY	PHT	EHT	AD	Response
0	0.94	0.45	−0.89	−0.45	−0.36	3.25
1	0.86	0.44	−0.88	−0.44	−0.12	2.99
2	0.75	0.41	−0.83	−0.41	−0.10	2.79
Mean1*^b^*	0.85	0.43	−0.87	−0.43	−0.19	3.01
Mean2*^c^*	—	0.29	−0.58	−0.29	−0.13	2.01

a Constrained Linear Genomic Selection Index.

b Mean1 is the average of the three selection cycles.

c Mean2 = Mean1/1.5, where 1.5 is the interval between selection cycles and denotes the average of the genetic gain per year.

**Table 3:** Two real datasets for estimated LGSI*^a^* parameters constructed with four traits (GY, PHT, EHT and AD) without constraints for three selection cycles

	Dataset 1				
	Estimated expected genetic gain per trait				Estimated maximum
Cycle	GY	PHT	EHT	AD	Response
0	0.23	−6.05	−6.45	−0.15	5.05
1	0.21	−5.15	−5.47	−0.07	4.29
2	0.18	−4.94	−5.60	−0.15	4.21
Mean1*^b^*	0.21	−5.38	−5.84	−0.12	4.52
Mean2*^c^*	0.14	−3.59	−3.89	−0.08	3.01
	Dataset 2				
	Estimated expected genetic gain per trait				Estimated maximum
Cycle	GY	PHT	EHT	AD	Response
0	0.33	−2.65	−2.47	−0.36	3.57
1	0.35	−1.73	−1.44	−0.17	2.85
2	0.29	−1.81	−1.61	−0.19	2.67
Mean1*^b^*	0.32	−2.06	−1.84	−0.24	3.03
Mean2*^c^*	0.22	−1.37	−1.23	−0.16	2.02

a Unconstrained Linear Genomic Selection Index.

b Mean1 is the average of the three selection cycles.

c Mean2 = Mean1/1.5, where 1.5 is the interval between selection cycles and denotes the average of the genetic gain per year.

Folder Simulated_Data_GSI contains two folders: Data_Phenotypes_April-26-15 and Haplotypes_GSI_April-26-15. In turn, folder Data_Phenotypes_April-26-15 contains two folders: GSI_Phenotypes-05 and PSI_Phenotypes-05. Within folder GSI_Phenotypes-05, there are six Excel data files, each denoted as C2_GSI_05_Pheno, C3_GSI_05_Pheno, C4_GSI_05_Pheno, C5_GSI_05_Pheno and C6_GSI_05_Pheno, corresponding to the simulated phenotypic information for the genomic selection index for cycles 2-7. In addition, folder GSI_Phenotypes-05 contains eight Excel datasets denoted as C0_Pheno_05, C1_PSI_05_Pheno, C2_PSI_05_Pheno, C3_PSI_05_Pheno, C4_PSI_05_Pheno, C5_PSI_05_Pheno, C6_PSI_05_Pheno, and C7_PSI_05_Pheno corresponding to the phenotypic simulated information for the phenotypic selection index for cycles 0-7. File Haplotypes_GSI_April-26-15 contains the haplotypes of the markers for cycles 0-7 of GSI. We present the results of the simulated datasets in Tables 4, 5 and 6.

**Table 4:** Simulated data for estimated CLPSI*^a^* and CLGSI*^b^* parameters constructed with four traits (T1, T2, T3 and T4), two constraints and true maximum responses in seven selection cycles

		CLPSI					True maximum
		Estimated expected genetic gains per trait				Estimated maximum	
Cycle	Theta	T1	T2	T3	T4	Response	Response
1	8.37	7.90	−3.16	3.55	1.70	16.31	19.51
2	7.53	7.48	−2.99	3.23	1.79	15.50	17.56
3	6.34	6.93	−2.77	2.74	1.65	14.08	16.49
4	7.19	7.75	−3.10	2.17	1.25	14.28	16.30
5	6.21	7.02	−2.81	2.46	1.33	13.62	15.96
6	4.92	6.30	−2.52	2.00	1.21	12.03	14.57
7	4.17	5.57	−2.23	2.58	1.17	11.54	14.65
Mean1*^c^*	6.39	6.99	−2.80	2.68	1.44	13.91	16.43
Mean2*^d^*	—	1.75	−0.70	0.67	0.36	3.48	4.11
		CLGSI					True maximum
		Estimated expected genetic gains per trait				Estimated maximum	
Cycle	Theta	T1	T2	T3	T4	Response	Response
1	6.13	6.83	−2.73	2.79	1.47	13.82	12.65
2	5.67	6.57	−2.63	2.69	1.41	13.29	15.27
3	5.26	6.28	−2.51	2.69	1.41	12.90	15.10
4	4.36	5.79	−2.32	2.29	1.21	11.60	16.03
5	3.78	5.37	−2.15	2.15	1.17	10.84	15.17
6	3.44	5.13	−2.05	1.92	1.21	10.31	14.28
7	3.78	5.39	−2.15	2.17	1.11	10.82	15.73
Mean1*^c^*	4.63	5.91	−2.36	2.38	1.28	11.94	14.89
Mean3*^e^*	—	3.94	−1.58	1.59	0.86	7.96	9.92

a Constrained Linear Phenotypic Selection Index;

b Constrained Linear Genomic Selection Index;

c Mean1 is the average of the seven selection cycles;

d Mean2 = Mean1/ 4 for the CLPSI;

e Mean3 = Mean1/1.5 for the CLGSI, where 4 and 1.5 are the intervals between selection cycles for CLPSI and CLGSI, respectively. Means 2 and 3 denote the average of the genetic gains per year.

**Table 5:** Simulated data for estimated CLPSI*^a^* and CLGSI*^b^* parameters constructed with four traits (T1, T2, T3 and T4), three constraints and true maximum responses in seven selection cycles

		CLPSI					True maximum
		Estimated expected genetic gains per trait				Estimated maximum	
Cycle	Theta	T1	T2	T3	T4	Response	Response
1	7.30	7.12	−2.85	4.27	1.55	15.79	17.47
2	6.43	6.61	−2.64	3.97	1.76	14.97	16.24
3	5.32	6.03	−2.41	3.62	1.52	13.58	15.15
4	4.45	5.54	−2.21	3.32	1.29	12.37	13.95
5	4.78	5.76	−2.30	3.45	1.28	12.80	14.28
6	3.62	4.96	−1.98	2.98	1.32	11.24	13.11
7	3.65	5.00	−2.00	3.00	1.23	11.24	13.14
Mean1*^c^*	5.08	5.86	−2.34	3.52	1.42	13.14	14.76
Mean2*^d^*	—	1.46	−0.59	0.88	0.36	3.28	3.69
		CLGSI					True maximum
		Estimated expected genetic gains per trait				Estimated maximum	
Cycle	Theta	T1	T2	T3	T4	Response	Response
1	4.43	5.50	−2.20	3.30	1.40	12.41	11.76
2	3.98	5.19	−2.08	3.11	1.42	11.80	13.40
3	3.92	5.15	−2.06	3.09	1.44	11.74	13.47
4	3.05	4.57	−1.83	2.74	1.13	10.28	14.11
5	2.72	4.30	−1.72	2.58	1.16	9.75	13.47
6	2.29	3.92	−1.57	2.35	1.15	8.99	13.30
7	2.57	4.18	−1.67	2.51	1.12	9.48	14.36
Mean1*^c^*	3.28	4.69	−1.87	2.81	1.26	10.63	13.41
Mean3*^e^*	—	3.12	−1.25	1.87	0.84	7.09	8.94

a Constrained Linear Phenotypic Selection Index.

b Constrained Linear Genomic Selection Index.

c Mean1 is the average of the seven selection cycles.

d Mean2 = Mean1/ 4 for the CLPSI.

e Mean3 = Mean1/1.5 for the CLGSI, where 4 and 1.5 are the intervals between selection cycles for CLPSI and CLGSI, respectively. Means 2 and 3 denote the average of the genetic gains per year.

**Table 6:** Simulated data for estimated LPSI*^a^* and LGSI*^b^* parameters constructed with four traits (T1, T2, T3 and T4) without constraints and true maximum responses for seven selection cycles

	LPSI					True maximum
	Estimated expected genetic gain per trait				Estimated maximum	
Cycle	T1	T2	T3	T4	Response	Response
1	10.42	−5.47	3.78	2.04	17.81	19.63
2	10.11	−4.35	3.68	2.00	15.69	17.56
3	9.91	−4.07	3.32	1.66	14.22	16.49
4	10.94	−4.31	2.57	1.42	14.34	16.32
5	10.60	−3.51	3.04	1.48	13.64	15.99
6	10.02	−3.54	2.53	1.37	12.04	14.69
7	8.77	−3.49	3.14	1.38	11.61	14.90
Mean1*^c^*	10.11	−4.11	3.15	1.62	14.19	16.51
Mean2*^d^*	2.53	−1.03	0.79	0.41	3.55	4.13
	LGSI					True maximum
	Estimated expected genetic gain per trait				Estimated maximum	
Cycle	T1	T2	T3	T4	Response	Response
1	6.60	−3.50	2.70	1.60	14.40	13.26
2	6.30	−3.40	2.60	1.50	13.91	15.28
3	6.10	−3.30	2.70	1.50	13.61	15.37
4	5.60	−3.10	2.30	1.30	12.30	16.05
5	5.20	−2.80	2.10	1.30	11.40	15.17
6	4.90	−2.60	1.90	1.30	10.61	14.49
7	5.20	−2.70	2.10	1.20	11.21	15.82
Mean1*^c^*	5.70	−3.10	2.30	1.40	12.49	15.06
Mean3*^e^*	3.8	−2.07	1.53	0.93	8.33	10.04

a Unconstrained Linear Phenotypic Selection Index.

b Unconstrained Linear Genomic Selection Index.

c Mean1 is the average of the seven selection cycles.

d Mean2 = Mean1/ 4 for the LPSI.

e Mean3 = Mean1/1.5 for the LGSI, where 4 and 1.5 are the intervals between selection cycles (L) for LPSI and LGSI, respectively. Means 2 and 3 denote the average of the genetic gains per year.

## Results

### Real data

#### The normality assumption:

**Figure 1:**
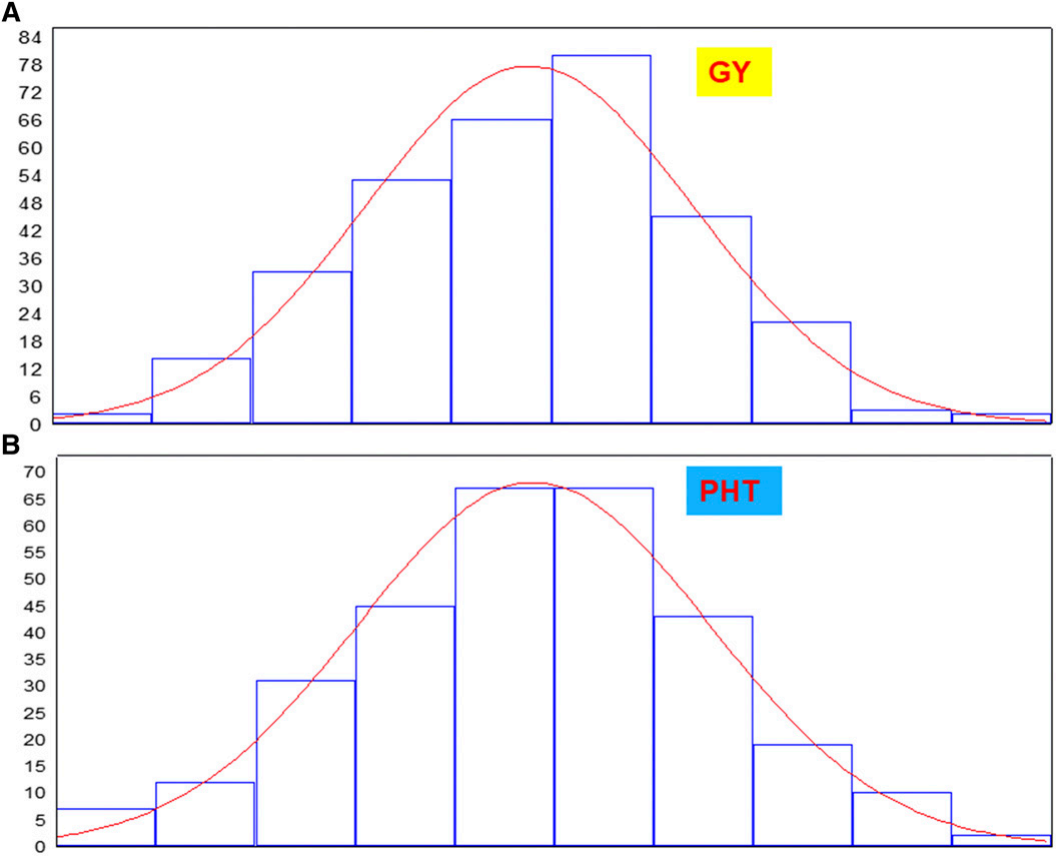
Distribution of the GEBVs (genomic estimated breeding values) associated with traits GY (Grain Yield, Fig. 1a) and PHT (Plant Height, Fig. 1b) for real Dataset 1 in cycle 1.

**Figure 2:**
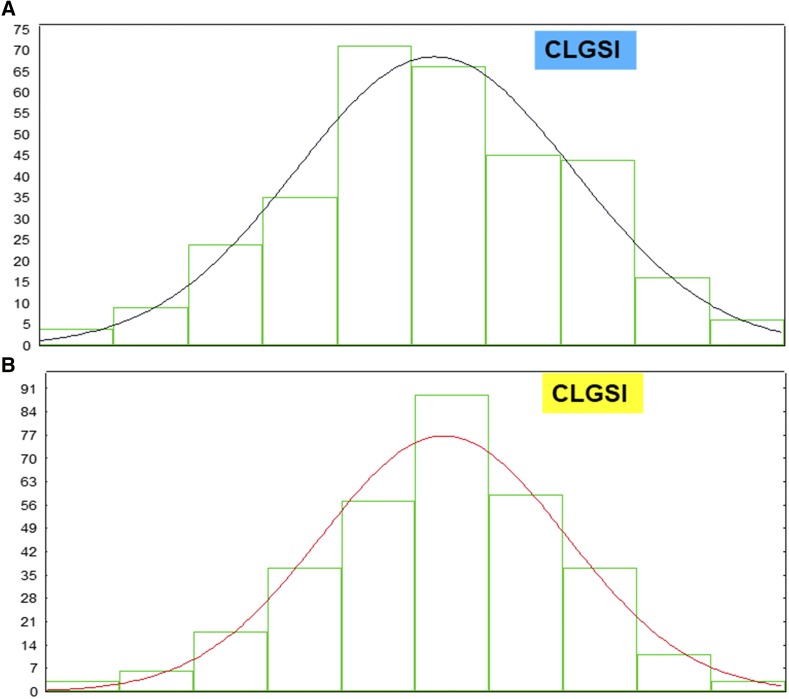
Distribution of the estimated CLGSI (constrained linear genomic selection index) values with two (Fig. 2a) and three (Fig. 2b) constraints for real Datasets 1 and 2, in cycles 1 and 2, respectively.

Figure 1 presents the frequency distribution of the GEBVs associated with traits GY (Figure 1a) and PHT (Figure 1b) for Dataset 1, in cycle 1. In addition, Figure 2 presents the frequency distribution of the CLGSI values for real Dataset 1 (in cycle 1) with two constraints (Figure 2a, $d^{\prime }=[\begin{array}{cc} 0.5 & -1.0 \end{array}]$), whereas Figure 2b presents the frequency distribution of the CLGSI values for real Dataset 2 (in cycle 2) with three constraints ($d^{\prime }=[\begin{array}{ccc} 0.5 & -1.0 & -0.5 \end{array}]$). Based on these results, we can assume that the GEBVs associated with traits GY and PHT, and the CLGSI values for the two set of restrictions, approach the normal distribution.

#### Estimated maximized CLGSI parameters:

Tables 2 and 3 present the estimated CLGSI and LGSI parameters, respectively. The estimated parameters are the theta ($\hat{\theta }$) values (for CLGSI only), the expected genetic gains per trait $\hat{E}$ (**Appendix 2**, Equation A10), and the selection responses $\hat{R}$ (**Appendix 2**, Equation A9). In Table 2, all parameters were estimated for datasets 1 and 2 with two ($d^{\prime }=[\begin{array}{cc} 0.5 & -1.0 \end{array}]$) and three ($d^{\prime }=[\begin{array}{ccc} 0.5 & -1.0 & -0.5 \end{array}]$) constraints, whereas in Table 3, the parameters were estimated, without constraints, for three selection cycles (0, 1 and 2). The estimated theta values were all positive (Table 2) for the two real datasets, which indicates that the CLGSI moves the population means in the desired direction, as we would expect.

#### Estimated CLGSI expected genetic gains per trait:

In Tables 2 and 3, there are two means: Mean1 and Mean2 = Mean1/*L* (*L=* interval between selection cycles) associated with the estimated CLGSI and LGSI expected genetic gain per trait and selection response for the two real maize (*Zea mays* L.) F2 populations, respectively. Mean1 is the average of the three selection cycles, whereas Mean2 (Mean1/1.5, where 1.5 is the interval between selection cycles or the time required to complete one selection cycle (Beyene *et al.* 2015)) is the average of the genetic gain per year.

For dataset 1, p=0.10, k=1.755 and two constraints, the estimated CLGSI expected genetic gains per trait for cycles 0, 1 and 2 were ${{\hat{E}}^{\prime }}_{0}=[\begin{array}{cccc} 0.76 & -1.51 & -4.05 & 0.71 \end{array}]$, ${{\hat{E}}^{\prime }}_{1}=[\begin{array}{cccc} 0.65 & -1.30 & -3.47 & 0.67 \end{array}]$ and ${{\hat{E}}^{\prime }}_{2}=[\begin{array}{cccc} 0.61 & -1.22 & -3.63 & 0.56 \end{array}]$, respectively. Each ${{\hat{E}}^{\prime }}_{q}$ (q= 0, 1, 2) value was associated with the mean values of traits GY, PHT, EHT and AD. Traits GY and PHT were constrained by $d^{\prime }=[\begin{array}{cc} 0.5 & -1.0 \end{array}]$ values. The Mean1 of the ${{\hat{E}}^{\prime }}_{q}$ value associated with traits GY and PHT (0.67 and -1.35, respectively) overestimated the $d^{\prime }$ values by 35% (Table 2). However, note that the genetic gain per year, obtained as Mean2 = Mean 1/1.5, underestimated the $d^{\prime }$ values by 10% because the Mean2 of the ${{\hat{E}}^{\prime }}_{q}$ values associated with traits GY and PHT (0.45 and -0.90, respectively) were lower than the $d^{\prime }$ values.

When we constrained three traits (GY, PHT and EHT) by vector $d^{\prime }=[\begin{array}{ccc} 0.5 & -1.0 & -0.5 \end{array}]$, we found results similar to those for two constraints. That is, the Mean1 of the ${{\hat{E}}^{\prime }}_{q}$ values associated with traits GY, PHT and EHT (0.75, -1.50 and -0.75, respectively) overestimated the $d^{\prime }$ values by 50% (Table 2). However, for Mean2 = Mean1/1.5, we found that the ${{\hat{E}}^{\prime }}_{q}$ values associated with traits GY, PHT and EHT (0.50, -1.0 and -0.50, respectively) were equal to the $d^{\prime }$ values. Thus, for dataset 1, the values of the vector of restrictions, $d^{\prime }=[\begin{array}{cc} 0.5 & -1.0 \end{array}]$ and $d^{\prime }=[\begin{array}{ccc} 0.5 & -1.0 & -0.5 \end{array}]$, were closer to Mean2 than to Mean1 values. This means that the estimated maximized expected genetic gain per trait estimated the genetic gain per year better than the genetic gain per selection cycle for dataset 1.

For dataset 2 (p=0.10 and k=1.755), with two ($d^{\prime }=[\begin{array}{cc} 0.5 & -1.0 \end{array}]$) and three ($d^{\prime }=[\begin{array}{ccc} 0.5 & -1.0 & -0.5 \end{array}]$) constraints, Mean1 for the ${{\hat{E}}^{\prime }}_{q}$ values associated with traits GY and PHT and those associated with GY, PHT and EHT, were closer to the $d^{\prime }$values than the Mean2 values (Table 2). In this case, the estimated expected genetic gain per trait estimated the genetic gain per year better than the genetic gain per selection cycle.

Dataset 1 gave higher estimated maximized CLGSI expected genetic gains per trait and genetic gains per year than dataset 2. This means that the number of genotypes affected the estimated values of the expected genetic gains (Table 1). The number of genotypes in real dataset 1 for cycles 0, 1 and 2 were 247, 320 and 303, respectively, whereas for dataset 2, the number of genotypes for those cycles were 181, 274 and 274 (Table 1), respectively.

#### Estimated maximized CLGSI selection response:

The results of the estimated maximized CLGSI selection responses for both datasets and constraints, were as follows. For dataset 1, the Mean1 of the estimated maximized CLGSI selection responses for two and three constraints were 4.24 and 4.29, respectively, while for dataset 2, those estimated values were 2.79 and 3.01. For dataset 1, the Mean2 of the estimated maximized CLGSI selection responses for two and three constraints were 2.82 and 2.99, respectively, while for dataset 2, those estimated values were 1.86 and 2.01. These results indicate that dataset 1 gave higher estimated CLGSI genetic gains per year. Again, we explain these results by noting that the number of genotypes in real dataset 1 for cycles 0, 1 and 2 were 247, 320 and 303, respectively, while for dataset 2, the number of genotypes for those cycles were 181, 274 and 274 (Table 1), respectively. This means that, in effect, the number of genotypes affected the estimated values of the selection response.

When we compared the CLGSI results (Table 2) with unconstrained LGSI results (Table 3), we found that the estimated CLGSI expected genetic gains per trait were different to the estimated unconstrained LGSI expected genetic gains per trait for both datasets. However, the estimated selection responses of both indices were very similar (Tables 2 and 3); this means that the two sets of constraints imposed on the CLGSI when we obtained its vector of coefficients mainly affected the CLGSI expected genetic gain per trait, as we would expect.

### Simulated data

#### Correlation of the GEBV With the true breeding values of the traits:

**Figure 3:**
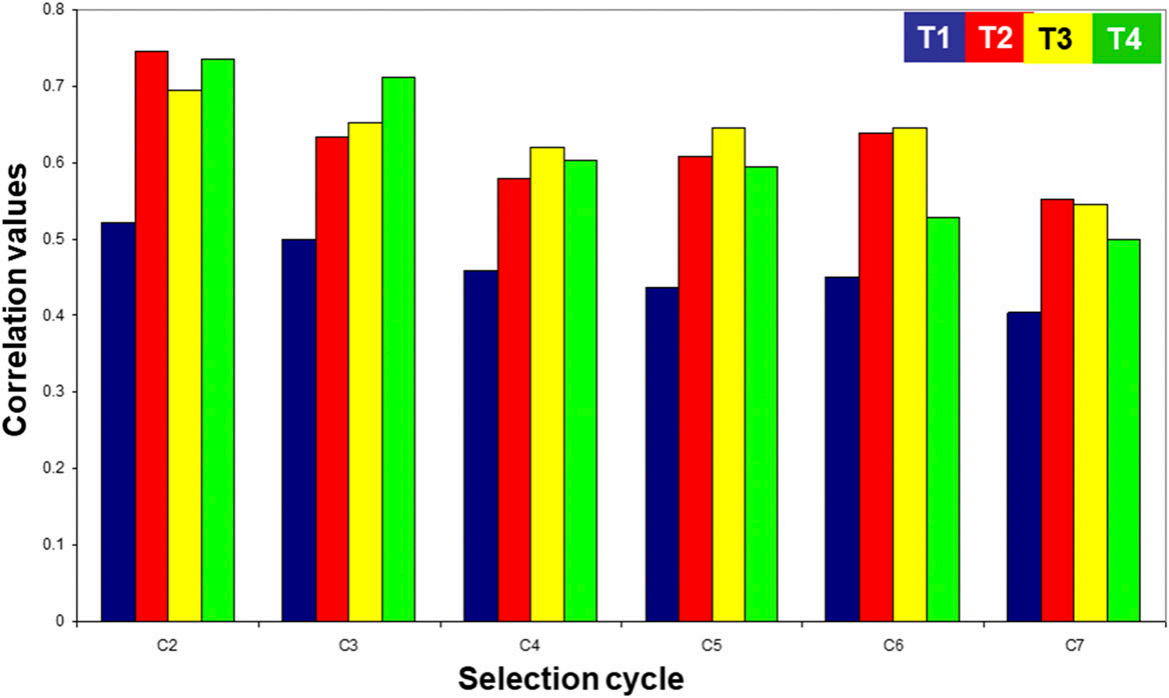
Correlations between the genomic estimated breeding values (GEBVs) and the true breeding values for four traits (T1, T2, T3 and T4) in six (C2 to C7) selection cycles. For each selection cycle, each column correspond to the correlations between the GEBV and the true breeding values for traits T1, T2, T3, and T4, respectively.

Figure 3 presents the estimated correlations between the GEBVs and true breeding values for four traits in six (C2 to C7) selection cycles. Each selection cycle contains four columns: the first column (from left to right) corresponds to the correlation between the GEBV and the T1 true breeding values; the second column corresponds to the correlation between the GEBV and the T2 true breeding values, and so on. In this figure, all correlation values tend to decrease. In C7, the correlation values between the GEBVs and the traits’ true breeding values were 0.40, 0.55, 0.54, and 0.50 for each of the four traits, respectively, whereas in cycle two (C2), these correlations were 0.52, 0.74, 0.69, and 0.73 for each of the four traits, respectively. In percentage terms, the correlation values of C7 were only 76%, 74%, 78%, and 68% of the correlation values in C2. That is, the correlation between the GEBVs and the traits’ true breeding values decreased more for traits 2 and 4 than for traits 1 and 3. We can explain these results by the number of QTL that affected each trait and the size of the QTL effects on the traits in each selection cycle. In all selection cycles, the estimated correlations were higher than or equal to 0.45; thus, the GEBVs obtained with the simulated data were good predictors of the individual breeding values, and so the CLGSI was a good predictor of the net genetic merit because the CLGSI is a linear combination of GEBV.

#### Estimated maximized CLPSI and CLGSI parameters:

Tables 4 and 5 present the estimated maximized CLPSI and CLGSI parameters, whereas Table 6 presents the unconstrained estimated maximized linear phenotypic and genomic selection index (LPSI and LGSI, respectively) parameters for seven simulated selection cycles. Céron-Rojas and Crossa (2018, Chapters 2 and 5) have given details of how to estimate the LPSI and LGSI parameters. We imposed two sets of constraints on the CLPSI and CLGSI expected genetic gains for seven selection cycles (C1 to C7). The vector for two constraints imposed on traits T1 and T2 was $d^{\prime }=[\begin{array}{cc} 5.0 & -2.0 \end{array}]$, whereas the vector for three constraints imposed on traits T1, T2 and T3 was $d^{\prime }=[\begin{array}{ccc} 5.0 & -2.0 & 3.0 \end{array}]$. The estimated theta values were all positive for the simulated dataset in the seven selection cycles for both sets of constraints, which indicates that CLPSI and CLGSI move the population means in the desired direction.

We shall compare the estimated maximized CLPSI parameters to the estimated maximized CLGSI parameters using the results in Tables 4 and 5. In Tables 4 and 5, there are three means: Mean1 and Mean2 (Mean3) = Mean1/*L* (*L=* interval between selection cycles) associated with the estimated maximized CLPSI and CLGCI expected genetic gain per trait and selection response, respectively. Mean1 is the average of the seven selection cycles, whereas, for the CLPSI, Mean2= Mean1/4 (4*=* interval between selection cycles, *L* (Beyene *et al.* 2015)) is the average genetic gain per year. For the CLGSI, *L* = 1.5 (Beyene *et al.* 2015), from where Mean3= Mean1/1.5. This means that the interval between selection cycles is a characteristic of each index and that the interval was shorter for the CLGSI than for the CLPSI.

The total proportion retained for both indices was p= 0.10 (k= 1.755) for all seven selection cycles. Each estimated CLPSI and CLGSI expected genetic gain per trait ${{\hat{E}}^{\prime }}_{q}$ (q=1, 2, …, 7) for two and three constraints is a vector of values associated with the expected genetic values of traits T1, T2, T3 and T4 (Equation 3). Some authors (Cerón-Rojas *et al.* 2016; Céeron-Rojas and Crossa 2018, Chapter 3) have described how to obtain ${{\hat{E}}^{\prime }}_{q}$ for the CLPSI. In **Appendix 2** (Equations A9 and A10), we describe how to estimate the maximized CLGSI selection response and expected genetic gain per trait.

#### Estimated and maximized CLPSI and CLGSI expected genetic gain per trait:

For two constraints ($d^{\prime }=[\begin{array}{cc} 5 & -2 \end{array}]$), the Mean1 of the estimated CLPSI expected genetic gain values associated with traits T1 and T2 were 6.99 and -2.80, respectively, whereas the Mean1 of the estimated CLGSI expected genetic gain values associated with traits T1 and T2 were 5.91 and -2.36, respectively. This means that the CLPSI overestimated the $d^{\prime }$ values by 38.80%, whereas the CLGSI overestimated those values by 18.20%.

For three constraints ($d^{\prime }=[\begin{array}{ccc} 5.0 & -2.0 & 3.0 \end{array}]$), the Mean1 of the estimated CLPSI expected genetic gain values associated with traits T1, T2 and T3 were 5.86, -2.34 and 3.52, respectively, whereas the Mean1 of the estimated CLGSI expected genetic gains associated with those traits were 4.69, -1.87 and 2.81, respectively. This means that while the CLPSI overestimated the $d^{\prime }$ values by 17.20%, the CLGSI underestimated those values by only 6.20%. Thus, the expected genetic gain per trait per selection cycle of the CLGSI was closer to the true gain than was the expected gain of the CLPSI. In addition, because the interval between selection cycles was higher for the CLPSI (*L* = 4) than for the CLGSI (*L* = 1.5), the genetic gain per year associated with the expected genetic gain per trait was also higher for the CLGSI than for the CLPSI (Tables 4 and 5).

Finally, note that the estimated LPSI and LGSI expected genetic gains per trait (Table 6) were more different from the vectors of constraints, than the estimated CLPSI and CLGSI expected genetic gains per trait. Thus, the two sets of constraints imposed on the CLGSI (CLPSI) when we obtained its vector of coefficients, affected the CLGSI (CLPSI) expected genetic gain per trait, as we would expect.

#### Efficiency of the CLPSI and CLGSI selection response:

In this subsection, we shall compare the average (Mean1) of the estimated CLPSI and CLGSI selection response to the true selection response obtained for both indices (Tables 4 and 5). In **Appendix 2** (Equations A11a and A11b), we indicate how we obtained the maximum true selection response for LPSI (LGSI) and for CLPSI (CLGSI). The maximum possible true selection responses for the economic index was the same for the genomic and phenotypic indices when we started the selection process, as this did not depend on the weights used in a particular selection index. However, from selection cycle two to selection cycle seven, the maximum possible true selection responses for both indices were different (Ceron-Rojas *et al.* 2015).

Again, let p= 0.10 (k=1.755) be the total proportion retained for both indices for all seven selection cycles with two set of constraints, as indicated in the last subsection. For two constraints, the average of the estimated CLPSI selection response (13.91) explained 84.66% of the true selection response (16.43), while the average of the estimated CLGSI selection response (11.94) explained only 80.0% of the true selection response (14.89) (Table 4). In a similar manner, for three constraints, the average of the estimated CLPSI selection response (13.14) explained 89.02% of the true selection response (14.76), while the average of the estimated CLGSI selection response (10.63) explained only 79.0% of the true selection response (13.41) (Table 5). That is, in this case, the estimated CLPSI selection response was closer to its true response than the estimated CLGSI selection response was to its true response. Note, however, that because the interval between selection cycles was higher for the CLPSI (*L* = 4) than for the CLGSI (*L* = 1.5), the genetic gain per year associated with the selection response was higher for the CLGSI than for the CLPSI (Tables 4 and 5) for both constraints.

Finally, when we compared the estimated CLPSI and LPSI selection responses, we observed that they were very similar. The same was true when we compared the estimated CLGSI and LGSI selection responses (Tables 4 and 6). This means that the two sets of constraints imposed on the expected genetic gains of both indices did not affect the selection response of the indices, at least for this dataset.

## Discussion

### CLGSI (CLPSI) expected genetic gain per trait and selection response

For the two real and simulated datasets, the CLGSI and CLPSI results indicated that CLGSI was more efficient than CLPSI per unit of time, but not per selection cycle, when the criterion for comparing the efficiency of both indices was the estimated selection response. However, when the criterion used to compare the efficiency of both indices was the estimated expected genetic gain per trait, the CLGSI was more efficient than the CLPSI per unit of time and per selection cycle. In addition, we found that when we compared the estimated CLPSI selection response with the LPSI selection response, they were very similar. The same was true for CLGSI and LGSI when we compared their selection responses. This means that the two sets of constraints imposed on the CLGSI (CLPSI) when we obtained its vector of coefficients, mainly affected the estimated and maximized CLGSI (CLPSI) expected genetic gain per trait, not its estimated selection response.

### Interval between selection cycles

For the two real and simulated datasets, the time required to conduct a selection cycle is a function of the time required to collect the data needed to estimate the index parameters. For CLPSI, the interval between selection cycles was 4, while for the CLGSI it was 1.5 (Beyene *et al.* 2015). The interval between selection cycles was the main factor that made CLGSI a more efficient index than CLPSI when we measured the efficiency of both indices by the genetic gains per year. It could also be the main justification for implementing genomic selection programs instead of phenotypic selection programs.

### Accuracy of the GEBV and the CLGSI values

For the simulated datasets, the average of the accuracy of the GEBV and the estimated correlation of the CLGSI with the net genetic merit were higher than or equal to 0.5. The accuracy of the GEBV in cycle 2 was 0.67, while in cycle 7 it was 0.5. The average of the estimated correlation of the CLGSI with the net genetic merit (data not presented) for the seven selection cycles was 0.8 for two constraints and 0.72 for three constraints. Thus, the correlation between the estimated CLGSI and the net genetic merit decreases when the number of constraints increased. Céron-Rojas and Crossa (2018; Chapters 5 and 6) have given a method for estimating the correlation of the CLGSI with the net genetic merit. In addition, Cerón-Rojas *et al.* (2016) found similar results in the CLPSI context. These results indicated the reliability of the GEBV for predicting the breeding value of the traits and of the CLGSI for predicting the net genetic merit; thus breeders can use the CLGSI as a good option for performing GS when there are genotyped individuals.

### The multivariate normality assumption

Based on the normality assumption of the estimated CLGSI (CLPSI) and GEBV values, we developed and applied the CLGSI (CLPSI) to real and simulated data. The histograms of the GEBV and the CLGSI values indicated that these values approached the normal distribution. The multivariate normality distribution is very important for breeding plant and animal quantitative traits because these traits show continuous variability and are the result of many gene effects interacting among themselves and with the environment. That is, quantitative traits are the result of unobservable gene effects distributed across plant or animal genomes, which interact among themselves and with the environment to produce the observable characteristic plant and animal phenotypes (Falconer and Mackay 1996). Under the multivariate normal distribution assumption, the traits under selection can be described using only means, variances, and covariances. In addition, if the traits are not correlated, they are independent; linear combinations of traits are also normal; and even when the trait phenotypic values do not have that distribution, the normal distribution serves as a useful approximation, especially in inferences involving sample mean vectors, which, by the central limit theorem, have multivariate normal distribution (Rencher 2002). By this reasoning, the fundamental assumptions in this work were that the GEBVs have multivariate normal distribution, while the net genetic merit and the index have bivariate normal distribution. Under the latter assumption, the regression of the net genetic merit on any linear function of the phenotypic values was linear.

### Criteria for evaluating the relative efficiency of the indices

We used four main criteria to compare CLGSI *vs.* CLPSI efficiency when performing genomic and phenotypic selection. Those criteria were the estimates of the theta values, the expected genetic gain per trait values, the selection response, and the interval between selection cycles. For real and simulated data, the estimated theta values were always positive, as we would expect. The estimated expected genetic gain per trait indicated how close these estimates were to the constraints imposed by the breeder for each trait, whereas the estimated selection response predicts the mean value of the net genetic merit in the progeny population. The interval between selection cycles is the time required to collect information to evaluate the index and complete one selection cycle. The four criteria were useful for evaluating and comparing the efficiency of both indices.

## Conclusion

We compared the relative efficiency of a CLGSI *vs.* a CLPSI. We determined the efficiency of both indices based on four criteria using real and simulated datasets. In both type of datasets, we found that the CLGSI genetic gain per year was higher than the CLPSI genetic gain per year because the CLGSI interval between selection cycles was shorter than the CLPSI interval. Therefore, breeders should use the CLGSI when performing selection.

## Appendix 1

### Genomic breeding values

In the training population, the *i*^th^ phenotypic value ($y_{i}$) can be denoted as $y_{i}=g_{i}+e_{i}$, where $g_{i}$ is the breeding value (*i.e.*, the average additive effects of the genes an individual receives from both parents) and $e_{i}$ is the residual. It is assumed that $g_{i}$ and $e_{i}$ are independent and that both have normal distribution with an expectation equal to zero and variance $\sigma_{g_{i}}^{2}$ and $\sigma_{e_{i}}^{2}$, respectively. The vector of genomic breeding values ${z^{\prime }}_{i}=[\begin{array}{cccc} z_{i1} & z_{i2} & \cdots & z_{in} \end{array}]$ associated with the vector of observations ${y^{\prime }}_{i}=[\begin{array}{cccc} y_{i1} & y_{i2} & \cdots & y_{in} \end{array}]$ (n= number of observations for trait i, i=1, 2,…,t; t= number of traits) of the candidates for selection for trait i can be written as

$$z_{i}=Xu_{i},$$ (A1)

where X is an n×m matrix (n=number of observations and m=number of markers in the population) of coded marker values (2−2q, 1−2q and −2q for genotypes *AA*, *Aa*, and *aa*, respectively) associated with the additive effects of the quantitative trait loci (QTL), and $u_{i}$ is an m×1 vector of the additive effects of the QTL associated with markers that affect the *i*^th^ trait. It is assumed that $z_{i}$ has multivariate normal distribution (MVN) with mean **0** and covariance matrix $G\sigma_{{\text{z}}_{i}}^{2}$, where $\sigma_{{\text{z}}_{i}}^{2}$ is the additive genomic variance of $z_{i}$ and $G=XX^{\prime }/c$ is the n×n additive genomic relationship matrix; $c=\sum_{j=1}^{m}2q_{j}(1-q_{j})$ in an F_2_ population; q is the frequency of allele *A* and 1−q is the frequency of allele *a* in the *j*^th^ marker (j=1,2,...,m).

Let $g_{ij}$ (i=1, 2,…,t, t=number of traits; j=1,2,...,n, n=number of observations) be the *j*^th^ element of the vector of true genotypic breeding values ${g^{\prime }}_{i}=[\begin{array}{cccc} g_{i1} & g_{i2} & \cdots & g_{in} \end{array}]$; then the covariance between $z_{ij}$ and $g_{ij}$ is $Cov(g_{ij},z_{ij})=\sigma_{z_{i}}^{2}$. That is, the covariance between $z_{ij}$ and $g_{ij}$ is equal to the additive genomic variance of $z_{ij}$ (Dekkers 2007). Let $z^{\prime }=[\begin{array}{cccc} z_{1} & z_{2} & \cdots & z_{t} \end{array}]$ and $g^{\prime }=[\begin{array}{cccc} g_{1} & g_{2} & \cdots & g_{t} \end{array}]$ be 1×t vectors of genomic and true breeding values, respectively, for t traits; then

Γ=Var(z)=Cov(z,g) (A2)

is the covariance matrix of genomic breeding values.

### Estimating marker effects

Let $u^{\prime }=[\begin{array}{cccc} {u^{\prime }}_{1} & {u^{\prime }}_{2} & \cdots & {u^{\prime }}_{t} \end{array}]$ be a vector 1×nt associated with t traits. The *i*^th^ vector $u_{i}$ (i=1, 2,…,t) of marker effects in the training population can be estimated as ${\hat{u}}_{i}=c^{-1}X^{\prime }{\left[G+\upsilon I_{n}\right]}^{-1}(y_{i}-1\mu_{i})$, where $\upsilon =\frac{\sigma_{e_{i}}^{2}}{\sigma_{g_{i}}^{2}}$; $\sigma_{g_{i}}^{2}$, $\sigma_{e_{i}}^{2}$, and the other parameters were defined earlier. In addition, we can estimate vector $u^{\prime }=[\begin{array}{cccc} {u^{\prime }}_{1} & {u^{\prime }}_{2} & \cdots & {u^{\prime }}_{t} \end{array}]$ in the multi-trait context as

$$\hat{u}=c^{-1}W^{\prime }{\left[\left(I_{t}⊗G)+(N⊗I_{n}\right)\right]}^{-1}(y-\mu ⊗1),$$ (A3)

where $W=I_{t}⊗X$, “⊗” denotes the Kronecker product (Schott 2005); c and X were defined in Equation (A1); $N=RC^{-1}$, R and C are the residual and genotypic covariance matrices for t traits, respectively; $y^{\prime }=[\begin{array}{cccc} {y^{\prime }}_{1} & {y^{\prime }}_{2} & \cdots & {y^{\prime }}_{t} \end{array}]$∼ MVN(μ, V) is a vector of size 1×tn, with covariance matrix $V=C⊗G+R⊗I_{n}$; $I_{t}$ and $I_{n}$ are identity matrix of size t×t and n×n, respectively; $\mu^{\prime }=[\begin{array}{cccc} \mu_{1} & \mu_{2} & \cdots & \mu_{t} \end{array}]$ is a vector 1×t of means associated with vector y, and 1 is a vector n×1 of 1's.

### Estimating genomic breeding values

We can estimate the vector $z^{\prime }=[\begin{array}{cccc} {z^{\prime }}_{1} & {z^{\prime }}_{2} & \cdots & {z^{\prime }}_{t} \end{array}]$ 1×nt of genomic breeding values for t traits in the testing population as

$$\hat{z}=W\hat{u}.$$ (A4)

Equation (A4) is the vector of GEBV for the multi-trait case and $W=I_{t}⊗X$ (Equation A3). Thus, in the testing population, the only thing that will change in Equation (A4) will be the coded values in matrix X, while $\hat{u}$ will be the same in each selection cycle. In Equations (A3) and (A4), we assumed that μ, C and R are known.

### Estimating the genomic covariance matrix Γ

Let ${\hat{z}}_{j}=X{\hat{u}}_{j}$ and ${\hat{z}}_{i}=X{\hat{u}}_{i}$ (Equation A4) be the genomic estimated breeding values (GEBVs) of $z_{j}=X_{i}u_{j}$ and $z_{i}=Xu_{i}$ (Equation A1), respectively, and denote by ${\hat{\mu }}_{j}$ and ${\hat{\mu }}_{i}$ the arithmetic means of the values of ${\hat{z}}_{j}$ and ${\hat{z}}_{i}$. We can estimate matrix Γ (Equation A2) when there is no phenotypic information, as

$$\hat{\Gamma }=\{{\hat{\sigma }}_{ji}\},$$ (A5)

where ${\hat{\sigma }}_{ji}=\frac{1}{g}({\hat{z}}_{j}-1{\hat{\mu }}_{j}{)}^{\prime }G_{}^{-1}({\hat{z}}_{i}-1{\hat{\mu }}_{i}{)}^{\prime }$ is the covariance between ${\hat{z}}_{j}=X_{i}{\hat{u}}_{j}$ and ${\hat{z}}_{i}=X{\hat{u}}_{i}$ values (j,i=1, 2, …, t); g is the number of genotypes; 1 is a g×1 vector of 1’s and $G=c^{-1}XX^{\prime }$ is the additive genomic relationship matrix (Ceron-Rojas *et al.*, 2015, for details).

## Appendix 2

### The CLGSI vector of coefficients

Let $D^{\prime }=\left[\begin{array}{ccccc} d_{r} & 0 & \ldots & 0 & -d_{1}\\ 0 & d_{r} & \ldots & 0 & -d_{2}\\ \vdots & \vdots & \ddots & \vdots & \vdots \\ 0 & 0 & \ldots & d_{r} & -d_{r-1} \end{array}\right]$ be a Mallard (1972) matrix (r−1)×r of constrained values, where $d_{i}$ is the *i*^th^ element of vector $d^{\prime }=[\begin{array}{cccc} d_{1} & d_{2} & \ldots & d_{r} \end{array}]$ (r is the number of traits constrained) of constrained values imposed by the breeder on the expected genetic gain of the trait (Equation 3), and let $U^{\prime }$ be a Kempthorne and Nordskog (1959) matrix (t−r)×t (t=number of traits) of 1’s and 0’s described in **Appendix 3**. To obtain the CLGSI vector of coefficients (β) that maximizes Equations (2) and (3), we will minimize the mean squared difference between $H=w^{\prime }g$ and $I=\beta^{'}z$ ($E[{\left(H-I\right)}^{2}]$) with respect to β under the restriction $D^{\prime }U^{\prime }\Gamma \beta =0$.

Let $M^{\prime }=D^{\prime }U^{\prime }\Gamma$ and suppose that Γ, $U^{\prime }$, $D^{\prime }$ and w are known. To minimize $E[{\left(H-I\right)}^{2}]$ under the restriction $M^{\prime }\beta =0$, we need to minimize the function

$$\Phi (\beta ,v)=\beta^{\prime }\Gamma \beta -2w^{\prime }\Gamma \beta +2v^{\prime }M^{\prime }\beta$$ (A6)

with respect to vectors β and $v^{\prime }=[\begin{array}{cccc} v_{1} & v_{2} & \cdots & v_{r-1} \end{array}]$, where v is a vector of Lagrange multipliers. Equation (A6) derivative results are: $\left[\begin{array}{cc} \Gamma & M\\ M^{\prime } & 0 \end{array}\right]\left[\begin{array}{c} b\\ v \end{array}\right]=\left[\begin{array}{c} \Gamma w\\ 0 \end{array}\right]$, from where the vector that minimizes $E[{\left(H-I\right)}^{2}]$ under the restriction $M^{\prime }\beta =0$ is

β=Kw, (A7)

where $K=[I_{t}-Q]$, $Q=UD{\left(D^{\prime }U^{\prime }\Gamma UD\right)}^{-1}D^{\prime }U^{\prime }\Gamma$ and $I_{t}$ is an identity matrix of size t×t. If in Equation (A7), $d_{i}=0$ for all traits, then D=U and the CLGSI will be similar to the Kempthorne and Nordskog (1959) index, but in the genomic selection context. When D=U and $U^{\prime }$ is a null matrix, β=w, the vector of economic weights or the unconstrained LGSI vector of coefficients (Ceron-Rojas *et al.*, 2015). Thus, the CLGSI is more general than the LGSI and includes the LGSI as a particular case.

### Estimating the CLGSI vector of coefficients

We can estimate β=Kw(Equation A7) as

$$\hat{\beta }=\hat{K}w,$$ (A8)

where $\hat{K}=[I_{t}-\hat{Q}]$, $\hat{Q}=UD{\left(D^{\prime }U^{\prime }\hat{\Gamma }UD\right)}^{-1}D^{\prime }U^{\prime }\hat{\Gamma }$. We defined all the parameters of Equation (A8) earlier.

### Estimating the maximized CLGSI selection response and expected genetic gain per trait

The estimated maximized CLGSI selection response (Equation 5) is

$$\hat{R}=k\sqrt{{{\hat{\beta }}^{\prime }}_{}\hat{\Gamma }\hat{\beta }},$$ (A9)

while the estimated optimized CLGSI expected genetic gain per trait (Equation 3) is

$$\hat{E}=k\frac{\hat{\Gamma }\hat{\beta }}{\sqrt{{{\hat{\beta }}^{\prime }}_{}\hat{\Gamma }\hat{\beta }}}.$$ (A10)

### Maximized true selection responses

We obtained the maximized true selection response for the unconstrained linear phenotypic and genomic selection indices (LPSI and LGSI, respectively) as

$$R_{P}=k\sqrt{w^{\prime }Cw}\;\text{and}\;R_{G}=k\sqrt{w^{\prime }\Gamma w},$$ (A11a)

respectively, where matrices C and Γ are the covariance matrix of true genotype values associated with the phenotypic (C) and the genomic values (Γ, Equation A2) in each selection cycle, k is the selection intensity and w is a vector of economic weights. In addition, the maximized true selection responses for CLPSI and CLGSI are

$$R_{P}=k\sqrt{b_{C}{}^{\prime }Cb_{C}}\;\text{and}\;R_{G}=k\sqrt{b_{\Gamma }{}^{\prime }\Gamma b_{\Gamma }},$$ (A11b)

respectively, where $b_{C}=K_{C}w$ and $b_{\Gamma }=K_{\Gamma }w$ are the vector of coefficients; $K_{C}=[I_{t}-Q_{C}]$, $K_{\Gamma }=[I_{t}-Q_{\Gamma }]$, $Q_{C}=UD{\left(D^{\prime }U^{\prime }CUD\right)}^{-1}D^{\prime }U^{\prime }C$, and $Q_{\Gamma }=UD{\left(D^{\prime }U^{\prime }\Gamma UD\right)}^{-1}D^{\prime }U^{\prime }\Gamma$ (Equation A7). Matrices C and Γ were defined in Equation (A11a). We obtained these last parameters only for the simulated datasets.

## Appendix 3

### The $U^{\prime }$ matrix of restrictions

In addition to matrix **D**, to obtain the CLGSI (CLPSI) vector of coefficients, we need the matrix $U^{\prime }$ (t−r)×t(t= number of traits; r=number of traits restricted), which is a matrix of 1’s and 0’s (1 indicates that the trait is restricted and 0 that the trait is not restricted), and depends on the number of restricted traits. Thus, suppose that we restrict only one of t traits; then, we can restrict the first of them as $U^{\prime }=[\begin{array}{ccccc} 1 & 0 & 0 & \cdots & 0 \end{array}]$, the second as $U^{\prime }=[\begin{array}{ccccc} 0 & 1 & 0 & \cdots & 0 \end{array}]$, the third as $U^{\prime }=[\begin{array}{ccccc} 0 & 0 & 1 & \cdots & 0 \end{array}]$, etc. When we restrict two of t traits, matrix $U^{\prime }$ could be constructed as follows. We can restrict the first and second traits as $U^{\prime }=\left[\begin{array}{ccccc} 1 & 0 & 0 & \cdots & 0\\ 0 & 1 & 0 & \cdots & 0 \end{array}\right]$, the first and third traits as $U^{\prime }=\left[\begin{array}{ccccc} 1 & 0 & 0 & \cdots & 0\\ 0 & 0 & 1 & \cdots & 0 \end{array}\right]$, the second and third traits as $U^{\prime }=\left[\begin{array}{ccccc} 0 & 1 & 0 & \cdots & 0\\ 0 & 0 & 1 & \cdots & 0 \end{array}\right]$, etc. If we restrict three of t traits, matrix $U^{\prime }$ will have the following form when the first, second and third traits are restricted: $U^{\prime }=\left[\begin{array}{cccccc} 1 & 0 & 0 & 0 & \cdots & 0\\ 0 & 1 & 0 & 0 & \cdots & 0\\ 0 & 0 & 1 & 0 & \cdots & 0 \end{array}\right]$; if the first, second and fourth traits are restricted, $U^{\prime }=\left[\begin{array}{cccccc} 1 & 0 & 0 & 0 & \cdots & 0\\ 0 & 1 & 0 & 0 & \cdots & 0\\ 0 & 0 & 0 & 1 & \cdots & 0 \end{array}\right]$, if the second, the third and the fourth traits are restricted, $U^{\prime }=\left[\begin{array}{cccccc} 0 & 1 & 0 & 0 & \cdots & 0\\ 0 & 0 & 1 & 0 & \cdots & 0\\ 0 & 0 & 0 & 1 & \cdots & 0 \end{array}\right]$, etc. The procedure used to construct matrix $U^{\prime }$ is valid for any number of restricted traits (Cerón-Rojas and Crossa 2018, Chapter 3, for details).
